# Indigenous microbiome as a key strategy for producing green chemicals

**DOI:** 10.3389/fmicb.2026.1798480

**Published:** 2026-03-27

**Authors:** Alejandra Martorell-Múgica, Cristina Gónzalez-Fernández, Silvia Greses

**Affiliations:** 1 Instituto IMDEA Energía, Móstoles, Spain; 2 Faculty of Chemical Sciences, Universidad Complutense de Madrid, Madrid, Spain; 3 Institute of Sustainable Processes, Universidad de Valladolid, Valladolid, Spain; 4 Department of Chemical Engineering and Environmental Technology, School of Industrial Engineering, Universidad de Valladolid, Valladolid, Spain; 5 CALAGUA – Unidad Mixta UV-UPV, Department of Chemical Engineering, Universitat de València, Valencia, Spain

**Keywords:** agroindustrial waste, ethanol, lactic acid, self-anaerobic fermentation, short-chain fatty acids

## Abstract

**Introduction:**

Reactors used for conventional anaerobic fermentation (C-AF) devoted to metabolite production are typically seeded with sludge collected from anaerobic digesters. This inoculum contains methanogens, which are the principal consumers of metabolites involved in biogas production. The use of the indigenous microbial community naturally present in the agroindustrial waste (AGW) was evaluated as an alternative inoculum to take advantage of its naturally scarce methanogenic abundance.

**Methods:**

Self-AF (lacking external inoculum) and C-AF were compared in terms of bioconversion yields and metabolite profiles. The effect of pH on community specialization and product distribution was assessed across the pH range naturally occurring during self-acidification.

**Results:**

Self-AF showed high bioconversion yields to metabolites (65.7 %) when compared to C-AF (58.7 %). Nevertheless, the inherent pH changes that the process suffered from self-acidification also resulted in metabolite profile oscillation. Whereas a pH of 4.5 maximized the lactic acid and ethanol production (13.9 and 11.7 g·L^−1^, respectively) due to the lactic acid bacteria prevalence, when a pH of 6 was reached, the microbiome specialized in carboxylates production, leading to a concentration of 29.8 g·L^−1^ in the steady state, with Clostridiales (51.8%) and Bifidobacteriaceae (21.4 %) as key bacteria.

**Conclusion:**

This study demonstrated the feasibility of conducting AF in the absence of external inoculum. Moreover, the wide bacterial metabolisms present in the indigenous microbiome revealed its capability of maximizing product portfolio using self-AF.

## 1 Introduction

In the last decades, the relentless expansion and development of the global population has led to an increase in food production and consumption, consequently raising environmental concerns related to resource exhaustion (water and nutrients) and waste production (Zhi Ling et al., 2023). It has been estimated that approximately one-third of all produced food is wasted on a global scale (FAO, 2019). This waste mainly consists of fruits and vegetables, representing 60% of all food supply chain (Sagar et al., 2018). In Europe, agriculture has been identified as the leading economic sector, producing over 37 million tons of agroindustrial waste (AGW) annually (Astudillo et al., 2023). Thus, managing these wastes becomes crucial, as their disposal or treatment following conventional technologies releases greenhouse gasses, aggravating global warming and climate change (Zhi Ling et al., 2023). In line with circular economy principles, new strategies for AGW valorization should be explored to circumvent the negative impacts while recovering valuable resources.

The valorization of AGW through biological processes has gained attention during the past years since soft conditions are normally required. Traditionally, the organic fraction (carbohydrates, proteins, and lipids) present in AGWs has been transformed into biogas (CO_2_ and CH_4_) through anaerobic digestion (AD) as the main bioprocess to valorize AGW into energy. AD consists of four main steps: hydrolysis, acidogenesis, acetogenesis, and methanogenesis. During the hydrolysis and acidogenesis steps (in the so-called anaerobic fermentation, AF), intermediate metabolites with high added value are also produced, including short-chain fatty acids (SCFAs), also named carboxylates, ethanol (EtOH), lactic acid (HLac), and hydrogen (H_2_) (Greses et al., 2022). Studies related to metabolite (SCFAs, EtOH, HLac, and H_2_) production through AF have been normally carried out using open-mixed cultures collected from conventional AD as inoculum. Although the high biodiversity of the AD microbiome exhibits a broad metabolic capacity to degrade complex organic matter (e.g., AGW), this type of inoculum also presents methanogens. This fact is a disadvantage when targeting those metabolites because methanogens are the main metabolite consumers for methane production purposes. Several strategies have been addressed to restrict methanogens’ activity, such as chemical deactivation using 2-bromoethanesulfonate and 2-mercaptoethanesulfonate (Logroño et al., 2022) or thermal pretreatments (Lin and Hung, 2008). However, those strategies present a downside since both the addition of reagents and the heat pretreatment may inhibit other microbes that produce metabolites (Wainaina et al., 2019). Alternatively, recent studies have demonstrated high AF performance by only tuning the operational parameters, without requiring costly pretreatments or chemical reagents. Magdalena et al. (2019) demonstrated that low process temperature has a significant effect on AF performance, being able to restrict methanogenic activity while maintaining high hydrolysis and acidogenesis activities. Moreover, Wainaina et al. (2019) determined that short hydraulic retention times (HRTs) enabled methanogen washout, thereby promoting fermentative bacterial enrichment. Nevertheless, the complete restriction of methanogenic activity by varying the operational conditions is still challenging. For instance, reducing the HRT may suppress methanogens, but this strategy can also compromise the hydrolysis yield when using highly particulate feedstock (Lv et al., 2022).

To address these challenges, this investigation proposed the use of indigenous microorganisms (IMOs) as inoculum for self-AF. The hypothesis hereby investigated dealt with the use of native microbiota from AGW to take advantage of a natural presence of AGW degraders alongside an intrinsically reduced methanogenic activity, thereby enhancing the production of high added-value metabolites. Although this type of consortium has been used for the production of HLac and H_2_ (Kim et al., 2009; Tang et al., 2016), its application for SCFA production has only recently started to be explored (Tang et al., 2023). Building upon these recent advances, this investigation aimed to evaluate the possibility of actively shaping the IMOs to maximize SCFA accumulation and reach a similar profile to the conventional AF. It is hypothesized that by applying specific operational controls (i.e., pH regulation), the microbial community composition can be steered to minimize SCFA-consuming taxa and promote a stable SCFA-producing consortium. To prove this hypothesis, the behavior of key microorganisms was monitored and correlated with metabolite output to achieve a comprehensive understanding of the microbiome development throughout the self-AF process and to evaluate whether its metabolic performance is comparable to that of conventional inoculated systems.

## 2 Materials and methods

### 2.1 Groindustrial waste used as feedstock

Cucumber, zucchini, and melon collected from market surpluses were selected as carbohydrate-rich feedstock to be valorized into added-value compounds through AF. These vegetables and fruit represent part of the largest AGWs generated in Spain (Tesfaw et al., 2021). Prior to subjecting AGW to biological valorization, the feedstock was mashed up and homogenized using a kitchen blender. As shown in Table 1 (average of *n* = 7 independent batches), AGW was chemically characterized in terms of total and soluble chemical oxygen demand (TCOD and SCOD, respectively), total and volatile solids (TS and VS), ammonium (NH_4_^+^-N), EtOH, HLac, SCFAs [acetic acid (HAc), propionic acid (HPro), butyric acid (HBu), valeric acid (HVal), and caproic acid (HCa)], glucose and fructose concentration, pH, and the content of carbohydrates, proteins, lipids, ash, and lignin.

**Table 1:** Chemical characterization of AGW used as feedstock.

Parameter	AGW
pH	4.6 ± 0.1
TCOD (g·L^−1^)	75.7 ± 5.1
SCOD (%)	81.0 ± 5.7
TS (g·L^−1^)	65.8 ± 4.6
VS (%)	90.7 ± 0.8
NH_4_^+^-N (g N·L^−1^)	0.08 ± 0.01
Carbohydrates (%)^*^	75.8 ± 0.7
Proteins (%)^*^	11.8 ± 1.3
Ash (%)^*^	9.2 ± 0.8
Lipids (%)^*^	8.7 ± 3.8
Lignin (%)^*^	4.8 ± 0.9
Glucose (g·L^−1^)	22.9 ± 3.4
Fructose (g·L^−1^)	24.2 ± 2.1
EtOH (g·L^−1^)	1.8 ± 0.4
SCFAs (g·L^−1^)	0.5 ± 0.2

Mean ± standard deviation (*n* = 7). ^*^Dry matter basis.

### 2.2 Anaerobic reactors: description and process performance

AF of AGW was carried out in two 1.5-L continuous stirred tank reactors (CSTRs) with a working volume of 1 L. One CSTR was inoculated with conventional AD sludge collected from an anaerobic digester located in a wastewater treatment plant (El Soto, Móstoles, Spain) to perform conventional AF (C-AF). In parallel, the growth of IMOs was promoted in another CSTR to evaluate the self-AF. To this end, the CSTR was initially filled with 1 L of AGW and incubated for 5 days in batch mode to provoke fermentative bacterial enrichment. The headspace (0.5 L) of each CSTR was connected to a flow meter (Ritter MGC1 V3.4, Germany) to measure the daily gas production. The CSTRs were homogenized using a magnetic stirrer (Agimatic-S, J. P. Selecta, S. A., Spain), and the temperature was maintained at 25 °C using a temperature-controlled water bath (F12-ED v2.0, Julabo, Germany). AFs were performed at an organic loading rate (OLR) of 3 g VS·L^−1^ d^−1^, corresponding to a hydraulic retention time (HRT) of 19.4 ± 1.5 d based on the VS composition of the AGW (Table 1). pH was daily monitored and adjusted to 6 by adding NaOH (6 M). In self-AF, the CSTR was subjected to three pH periods in which the pH was not initially controlled (start-up), underwent progressive pH adjustment (transition), and reached a final pH of 6 (steady state). These conditions were selected according to previous studies in which high OLR, 25 °C, and slightly acidic pH resulted in high bioconversion efficiencies when performing conventional AF by adding external inoculum (i.e., anaerobic sludge) (Greses et al., 2020).

Steady state of the AF processes was determined when the reactor operation exceeded three HRTs (3 × 20 d) and both effluents (gas and liquid) exhibited stability. AF performance was evaluated based on the average of independent samples collected during these steady-state periods, which lasted 21 days for C-AF (*n* = 7) and 35 days for self-AF (*n* = 11), ensuring the reliability of the data acquired. The process efficiency was assessed throughout the AF in terms of COD removal and bioconversion, in accordance with the Equations 1, 2. Hydrolysis and acidogenesis efficiencies in AF were also evaluated by calculating the VS removal (Equation 3) and acidification (Equation 4) percentages, respectively. In addition, COD mass balance (Equation 5) was evaluated following Elreedy et al. (2016) equation.


$$\%{\mathrm{COD}}_{\text{removal}}=\frac{{\text{TCOD}}_{\text{in}}-{\text{TCOD}}_{\text{out}}}{{\text{TCOD}}_{\text{in}}}\cdot 100$$ (1)

$$\%\text{Bioconversion}=\frac{{\mathrm{COD}}_{\text{metabolites}}}{{\text{TCOD}}_{\text{in}}}\cdot 100$$ (2)

$$\%{\mathrm{VS}}_{\text{removal}}=\frac{{\mathrm{VS}}_{\text{in}}-{\mathrm{VS}}_{\text{out}}}{{\mathrm{VS}}_{\text{in}}}\cdot 100$$ (3)

$$\%\text{Acidification}=\frac{{\mathrm{COD}}_{\text{metabolites}}}{{\text{SCOD}}_{\text{out}}}\cdot 100$$ (4)

$$\%\mathrm{COD}\;\text{mass balance}=\frac{{\text{TCOD}}_{\text{out}}+{\mathrm{COD}}_{\mathrm{gas}}\;}{{\text{TCOD}}_{\text{in}}}\cdot 100$$ (5)

TCOD_out_ indicates the total organic matter measured in the CSTR effluent, and TCOD_in_ represents the total organic matter determined in the AGW in terms of g COD·L^−1^. COD_metabolites_ represents the content of metabolites (HLac, EtOH, and SCFAs) in the CSTR effluent measured as g COD·L^−1^ equivalent, which are 1.066 for HLac, 2.285 for EtOH, 1.066 for HAc, 1.513 for HPro, 1.820 for HBu, 2.039 for HVal, and 2.207 for HCa. VS_in_ and VS_out_ represent the g VS·L^−1^ in the AGW and effluent of CSTR, respectively. SCOD_out_ is the soluble organic matter content determined in the CSTR effluent in terms of g COD·L^−1^. COD_gas_ corresponds to the COD equivalents (g COD·L^−1^) of the gaseous products [methane (CH_4_) and hydrogen (H_2_)].

To objectively quantify process stability during the steady state described above, a stability index (SI) was calculated with the mean (X̅) and standard deviation (std x) of the parameter values [pH, solids (TS and VS), chemical oxygen demand (TCOD and SCOD), N-NH_4_^+^, gas, and metabolites] during the steady state according to Tenca et al. (2011)


$$\mathrm{SI}=\frac{\mathrm{std}\;x}{\bar{x}}\cdot 100$$ (6)

Based on Equation 6, SI values near 1.0 indicated minimal fluctuation, while SI values > 0.7 were considered representative of stable operation (Tenca et al., 2011).

### 2.3 Analytical methods

For the substrate characterization, TS, VS, and ash content were regularly measured according to the methods 2,540 B (TS) and 2,540 E (VS and ash), which are defined in Standard Methods (APHA, 2017). Carbohydrate content was analyzed following the phenol-sulfuric acid procedure described by Dubois et al. (1956). Total Kjeldahl nitrogen (TKN) was measured by applying the method 4,500-N_org_ detailed in Standard Methods (APHA, 2017), and protein content was determined by multiplying TKN by a conversion factor of 6.25 (Hickert et al., 2013). Lipid percentage was obtained by calculating the difference between 100 and the content of carbohydrates, proteins, and ash. The glucose and fructose concentrations in the AGW feedstock were measured by liquid chromatography in an Agilent 1,260 HPLC-RID (Agilent, United States) equipped with a pre-column (CARBOSep CHO-682 LEAD Column, Transgenomic) and an ion exchange column (CARBOSep CHO 682 Pb, 7.8 ⋅ 300 mm, Concise) operating at 80 °C, using Milli-Q water as the mobile phase and conducting the elution at a flow rate of 0.4 mL·min^−1^. Detection was performed by a refractive index detector (RID) working at 35 °C. Lignin content was analyzed following the National Renewable Energy Laboratory (NREL) standard methods for the determination of structural carbohydrates and lignin in the biomass (LAP-001, LAP-002, LAP-003, LAP-004, and LAP-019). TCOD, SCOD, and NH_4_^+^-N were measured in the AGW periodically. To obtain the soluble fraction, samples were filtered by a 0.45-μm nylon filter. The analysis of those compounds was conducted through colorimetric quantification following Standard Methods 5220D (TCOD and SCOD) and 4500F-NH_3_ (NH_4_^+^-N) (APHA, 2017). The concentration of metabolites (HLac, EtOH, and SCFAs) in the influent was analyzed using liquid chromatography after filtering the samples through a 0.22-μm nylon filter and using an Agilent 1,260 HPLC-RID (Agilent, United States) equipped with a pre-column (Cation H Refill Cartridge Microguard column, Biorad) and an ion exclusion column (Aminex HPX-87H 300⋅ 7.8 mm I. D., Biorad). The mobile phase used was 5 mM H_2_SO_4_, and elution was conducted at a flow rate of 0.6 mL·min^−1^. The injection volume was 20 μL, and the oven temperature was 44 °C. The detection was conducted by an RID operating at 35 °C.

TS, VS, ash, TCOD, SCOD, NH_4_^+^-N, and concentration of metabolites were also determined twice per week in the CSTR effluents, following the methods aforementioned. pH was daily monitored in AF effluents using a pH meter GLP 21 (Crison, Hach Lange). Biogas composition (CH_4_ and H_2_) in the headspace of the CSTR was determined twice per week using a gas chromatograph (GC) fitted with a thermal conductivity detector (Clarus 580 GC, PerkinElmer) and equipped with two coupled packed columns (HSN6-60/80 Sulfinert P 7′ ⋅ 1/8″ O. D. and MS13X4-09SF2 40/60 P 9′ ⋅ 1/8″ O. D., PerkinElmer). Biogas analysis was conducted according to the procedure specifications defined by Greses et al. (2022). The limits of detection (LODs) for the GC were calculated according to the International Council for Harmonization (ICH) guidelines (ICH, 2005). Based on their methodology, the LODs for the biogas components (CH_4_ and H_2_) were determined to be 1.18% (v/v) and 0.28% (v/v), respectively.

### 2.4 Microbial community analysis

To assess the effect of IMOs on the valorization of this residue into metabolites, microbial communities were identified throughout the process in both CSTRs, C-AF, and self-AF. To this end, 1-mL sample was collected weekly from the CSTRs and frozen at −80 °C. DNA extraction was carried out using FastDNA SPIN kit for soil (MP Biomedicals, LCC) following the manufacturer’s protocol. Quality and amount of the DNA were verified using a NanoDrop spectrophotometer (SPECTROstar Omega—BMG Labtech, DE) by measuring the 260/280 ratio and the concentration of the extracted DNA (ng·mL^−1^). The samples were sequenced by FISABIO (Valencia, Spain) on a MiSeq Sequencer (Illumina) using the primers 341F and 805R (F - CCTACGGGNGGCWGCAG and R - GACTACHVGGGTATCTAATCC), which targeted the hypervariable regions V3 and V4 of the 16S rRNA gene for both bacteria and archaea. Aiming at identifying the microorganisms present in the samples, the retrieved sequences were bioinformatically treated following the method detailed by Greses et al. (2017). Briefly, the sequence reads were subjected to a pre-processing pipeline prior to clustering using independent tools for merging (PEAR), quality filtering (PRINSEQ), and primer trimming and chimera removal (mothur). Since the aim of the present investigation was to evaluate broad microbial population shifts, QIIME 1.9.1 (Caporaso et al., 2010) was solely used on the resulting cured dataset for the final clustering into operational taxonomic units (OTUs) at 97% identity and the calculation of the Shannon index. The Bray–Curtis similarity matrix was used to measure microbial dissimilarities using the similarity percentages test (SIMPER). The SIMPER analysis and canonical correspondence analysis (CCA) were conducted using PAST 4.17 software (Hammer et al., 2001).

## 3 Results and discussion

### 3.1 Effect of inoculum on AF performance

Once the process reached the steady state, both CSTRs attained similar hydrolytic (46.8 and 49.3% of VS removal for C-AF and self-AF, respectively) and acidification efficiencies (95.9% in C-AF and 100.0% in self-AF) (Table 2). These values were in the range of previous studies related to conventional AF of AGW, representing high AF efficiencies. For instance, Gonçalves et al. (2024) reported a VS removal of 51% and acidification of 92.7% when conducting AF of AGW at 25 °C and pH 6.2 with the external addition of conventional anaerobic inoculum, concluding that fermentative metabolisms were promoted. Therefore, these results evidenced that IMOs presented a high fermentative activity, which was comparable with those values reached when external anaerobic bacteria were added.

**Table 2:** Composition of the CSTR effluents and process efficiencies when the AF reached the steady state using conventional sludge as inoculum (C-AF) and in the absence of external inoculum (self-AF).

Parameter	C-AF	Self-AF
pH	5.9 ± 0.2	6.0 ± 0.2
TCOD (g·L^−1^)	55.3 ± 1.0	66.7 ± 0.1
SCOD (% w/w)	70.3 ± 3.2	73.0 ± 5.1
TS (g·L^−1^)	45.1 ± 1.5	48.9 ± 1.9
VS (% w/w)	62.9 ± 1.0	59.0 ± 1.2
NH_4_^+^-N (g N·L^−1^)	0.4 ± 0.1	0.3 ± 0.1
Total metabolites (g·L^−1^)	25.5 ± 0.6	29.8 ± 0.7
HAc (% w/w)	25.8 ± 2.3	29.4 ± 3.3
HPro (% w/w)	2.9 ± 0.4	3.8 ± 0.3
HBu (% w/w)	54.2 ± 1.0	40.5 ± 2.0
HVal (% w/w)	8.3 ± 0.2	11.7 ± 2.5
HCa (% w/w)	7.0 ± 1.4	12.5 ± 0.6
EtOH (% w/w)	<LD^*^	<LD^*^
HLac (% w/w)	<LD^*^	<LD^*^
H_2_ (mL·g COD_in_^−1^)	<LD^*^	1.2 ± 0.9
CH_4_ (mL·g COD_in_^−1^)	27.5 ± 6.9	<LD^*^
Bioconversion metabolites (%)	58.7 ± 2.2	65.7 ± 0.9
Bioconversion SCFAs (%)	58.3 ± 2.5	65.6 ± 1.7
Bioconversion EtOH (%)	<LD^*^	<LD^*^
Bioconversion HLac (%)	<LD^*^	<LD^*^
COD removal (%)	24.3 ± 1.7	15.4 ± 1.5
Acidification (%)	95.9 ± 4.6	100.0 ± 4.9
VS removal (%)	46.8 ± 4.9	49.3 ± 3.3

*Lower than the limit of detection (LD). Mean ± standard deviation (*n* = 7 for C-AF and *n* = 11 for self-AF). Values of the stability index (SI) for each parameter are reported in Supplementary Table S3.

Despite the similarities in hydrolysis and acidification efficiencies, a variation was observed in the bioconversion efficiencies to metabolites. Whereas C-AF achieved 58.3% bioconversion, 66.9% was obtained in self-AF. The difference corresponded to a different total metabolite production of 25.5 g·L^−1^ for C-AF and 29.8 g·L^−1^ for self-AF (Table 2). This fact might be related to the presence of microorganisms involved in carboxylate consumption, such as syntrophic acetate-oxidizing bacteria (SAOB) and methanogens. Those microorganisms exhibit a synergy in conventional AD, which is essential to successfully produce biogas since SAOB transform HAc into H_2_ and CO_2_, while methanogens consume HAc or H_2_ to produce CH_4_ (Ziemiński and Fraç, 2012). Considering that C-AF was inoculated with sludge collected from AD, the likely presence of SAOB and methanogens could justify the lower bioconversion attained in this reactor. This was, in fact, confirmed by the COD removal determined in C-AF. As shown in Table 2, C-AF reached higher COD removal (24.3%) than self-AF (15.4%). Similarly, previous studies performing C-AF at comparable conditions (25 °C, OLR 3 g VS L^−1^ d^−1^, pH 6.1) reported a bioconversion of 59.6 and 20.1% of COD removal (Greses et al., 2023). These results showed a reproducible behavior to that attained when bacteria collected from AD were used as inoculum. It was confirmed that, even without the addition of external bacteria, the IMOs associated with the feedstock were able to successfully carry out AF. This highlighted a promising strategy to maximize metabolite production through AF, as the absence of metabolite-consuming microorganisms favors metabolite accumulation.

It is important to point out that, although self-AF evidenced a better performance than C-AF, both processes exhibited high bioconversion yields (> 50%). These results were supported by the proper selection of the operational conditions and the high carbohydrate content of the AGW (75.8%) (Table 1). It should be noted that these carbohydrates were easily biodegradable since the content of sugars, such as glucose and fructose, was also high (22.9 and 24.2 g·L^−1^, respectively). These sugars represent readily biodegradable organic matter. By contrast, the presence of recalcitrant compounds, such as lignin, represented a very low fraction in AGW (4.8%) (Table 1). These features enabled efficient hydrolysis and acidogenesis by both microbial cultures, justifying the outstanding efficiencies (Table 2).

Regarding the metabolite profile at the steady state, HAc and HBu were the predominant SCFAs in both CSTRs. The slight HCa increase in self-AF (12.5% w/w, 3.8 g·L^−1^) compared to C-AF was the most significant change in the metabolite profile, showing a remarkable similarity between the reactor’s effluents and confirming that external inoculum addition was not required to attain a targeted metabolite pool. This metabolite profile has been commonly observed when carbohydrate-rich residues were used as feedstock in AF at slightly acidic pHs (Greses et al., 2020). It could be thus confirmed that conducting AF at pH 6 of residues with a high content of carbohydrates, such as AGW, enabled the accumulation of even-chain carboxylic acids (HAc, HBu, and HCa).

**Figure 1:**
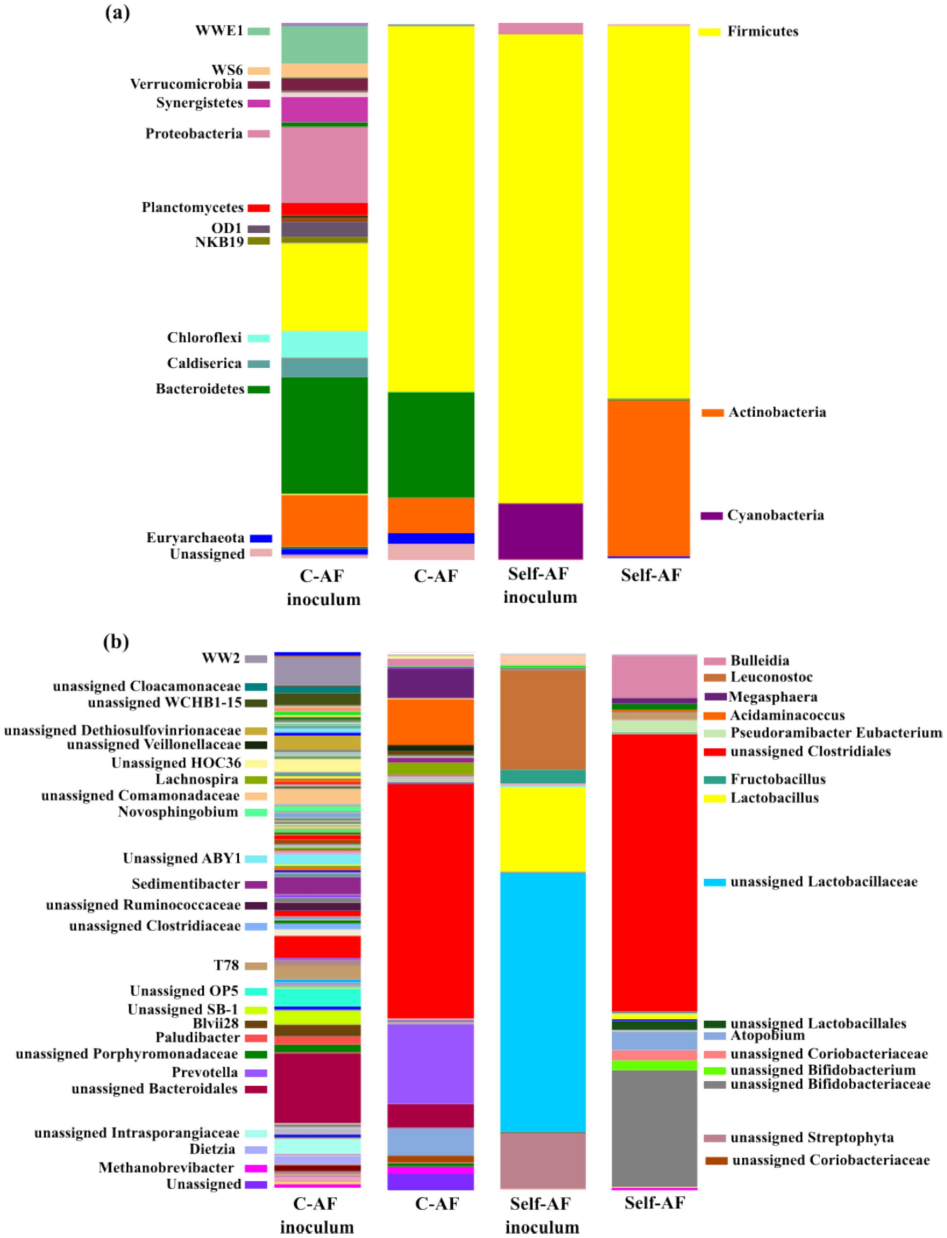
Bacterial and archaeal profiles were determined in the inocula and the steady state (C-AF and self-AF) at **(a)** phylum and **(b)** genus levels. Microorganisms with a relative abundance lower than 1% have been excluded from the legend (for family-level resolution, see Supplementary Figure S4).

The microbiome analysis revealed a remarkably higher biodiversity in C-AF (1,301 OTUs, Shannon index: 7.501) than that of self-AF (292 OTUs, Shannon index: 2.728). This fact was confirmed by the identification of those microorganisms in C-AF, such as Bacteroidetes and Euryarchaeota phyla (Figure 1a). Previous studies determined that Bacteroidetes encompass SAOB (Pan et al., 2021), with *Prevotella* identified as common bacteria in AD with high syntrophic acetate-oxidizing activity (Ruiz-Sánchez et al., 2018). C-AF presented a 32.5% relative abundance of *Prevotella*, along with a 1.9% of *Methanobrevibacter* (Euryarchaeota) (Figure 1b). *Methanobrevibacter* is known as hydrogenotrophic archaea that synergistically grow with SAOB (Ruiz-Sánchez et al., 2018). The coexistence of these taxa aligned with the methane yield observed in the C-AF reactor (27.5 mL CH_4_·g COD_in_^−1^) and the presence of SCFA consumers, explaining its lower bioconversion to SCFAs and higher overall COD removal (Table 2) when compared to self-AF. In the C-AF, the COD mass balance reached a closure of 83.4%, with the unclosed fraction likely associated with the slight methane production and the experimental deviations related to the high content of particulate organic matter. By contrast, self-AF was dominated by Firmicutes and Actinobacteria phyla, while Euryarchaeota and Bacteroidetes were not significantly detected (Figure 1a). Indeed, *Methanobrevibacter* (0.3%) was markedly limited in this reactor. Consistent with this restricted methanogenic population, CH_4_ production in the self-AF reactor was consistently remaining below the detection limit, explaining the reduced COD removal attained. Consequently, out of an influent load of 4.1 g COD_in_·d^−1^, the carbon flux in the self-AF reactor was primarily retained in the liquid phase as SCFAs (2.6 g COD·d^−1^), reaching a COD mass balance closure of 90.6%.

SIMPER analysis determined a 45.2% dissimilarity, mainly attributed to Bifidobacteriaceae (Actinobacteria phylum) in self-AF and *Prevotella* (Bacteroidetes) in C-AF (Supplementary Table S1). Bifidobacteriaceae have been described as heterofermentative bacteria involved in the hydrolysis and acidogenesis steps, producing both HAc and HLac from carbohydrates, including simple sugars (Cieciura-Włoch et al., 2020), such as those determined in AGW (glucose and fructose). The high HAc concentration attained in self-AF (Table 2) could be related to the production of HAc by Bifidobacteriaceae and also likely associated with the further conversion of HLac into HAc, since HLac was not detected in self-AF. This conversion was supported by the enrichment of Clostridiales, which have been reported to metabolize sugars and HLac to produce HBu and HAc (Sun et al., 2023). The accumulation of HAc (29.4% of the total metabolite pool) and HBu (40.5%) (Table 2) can thus be related to the high relative abundance of this genus (51.8%) (Figure 1b). Similarly, *Bulleidia* have also been described as fermentative bacteria involved in sugar degradation into SCFAs (Gulhane et al., 2017), which was in agreement with the high SCFA concentration in self-AF as related to their relative abundance of 20.6%.

Clostridiales was identified in C-AF (42.3%), along with *Megasphaera* and *Acidaminococcus*, which are hypothesized to exhibit a similar metabolic behavior to *Bulleidia* in self-AF (Ruiz-Sánchez et al., 2018).

To statistically validate these observations, a CCA was performed (Supplementary Figure S3). The first three axes of the CCA cumulatively explained 83.8% of the total variance (CCA 1—39.1%, CCA 2—25.6%, and CCA 3—19.1%). Along the CCA 1, the steady-state samples of C-AF and self-AF clustered together in the negative axis, exhibiting strong spatial correlation with the SCFA vectors of HAc, HBu, HVal, and HCa. This result confirmed a similar tendency toward secondary fermentation, supporting a functionally redundant association of SCFA-producing taxa, mainly represented by unassigned Clostridiales. Supplementary Figure S3b successfully captures the divergence between the two operational strategies. Specifically, the C-AF steady state was clearly separated into the negative axis of CCA 3, distinctively associated with *Prevotella*, *Acidaminococcus*, *Lachnospira*, *Megasphaera*, and the HPro vector. Notably, *Methanobrevibacter* was also positioned in this quadrant near the C-AF steady-state sample, confirming the active enrichment of a methanogenic metabolism. Conversely, the self-AF steady state was distinctly positioned on the positive side of CCA 3, which was strongly correlated with HCa accumulation and the enrichment of *Bifidobacterium* and *Bulleidia*. Moreover, *Methanobrevibacter* was positioned far from the self-AF steady-state sample (Supplementary Figure S3a) and negatively correlated within the CCA 3 axis (Supplementary Figure S3b), confirming the reduced methanogenic activity in this reactor and a more efficient carbon flux toward SCFA production.

These results evidenced that a similar metabolic profile can be reached in self-AF, with the higher bioconversion efficiency related to the absence of SCFA consumers by taking advantage of the microbiome developed under anaerobic conditions of IMOs.

### 3.2 Microbiome versatility

In addition to the advantage of using native microbiomes to maximize SCFA production, this microbiome can also be shaped to obtain other value-added compounds. A careful monitoring of self-AF response at microbiological and chemical levels evidenced the high metabolic versatility of IMOs subjected to pH variations. As shown in Supplementary Figure S1, the metabolite profile revealed the critical effect of pH on self-AF behavior. Based on the metabolic response against pH, self-AF evolution was divided into three periods (start-up, transition, and steady state).

#### 3.2.1 Start-up period

A pH drop was observed during the start-up period due to the inherent acidification of the AGWs, reaching a pH of 4.5 (Table 3). This fact resulted in an acidification yield of 99.0% and a VS removal of 52.3% (Table 3), confirming the high fermentative activity of the bacteria naturally present in the feedstock.

**Table 3:** Composition of the CSTR’s effluent and process efficiencies throughout the AF (mean ± standard deviation).

Period	Start-up		Transition	
Experimental day	3 (*n* = 2)	17 (*n* = 2)	24 (*n* = 2)	35 (*n* = 2)
pH	4.5 ± 0.1	5.9 ± 0.1	6.0 ± 0.1	6.0 ± 0.1
TCOD (g·L^−1^)	60.0 ± 1.1	67.3 ± 2.6	68.1 ± 1.1	64.3 ± 0.4
SCOD (% w/w)	75.4 ± 4.5	77.8 ± 2.0	79.8 ± 1.6	72.8 ± 3.4
TS (g·L^−1^)	34.6 ± 0.3	50.6 ± 0.9	42.0 ± 2.3	48.6 ± 3.2
VS (% w/w)	75.3 ± 0.1	58.8 ± 0.1	59.1 ± 1.3	57.4 ± 0.1
NH_4_^+^-N (g N·L^−1^)	0.1 ± 0.1	0.3 ± 0.1	0.5 ± 0.1	0.4 ± 0.1
Total metabolites (g·L^−1^)	28.4 ± 1.3	35.4 ± 3.8	31.5 ± 0.2	32.0 ± 0.3
HAc (% w/w)	7.3 ± 2.2	12.2 ± 4.0	15.8 ± 1.7	36.2 ± 0.8
HPro (% w/w)	0.8 ± 0.4	4.5 ± 10.7	17.6 ± 2.0	13.1 ± 2.7
HBu (% w/w)	<LD^*^	<LD^*^	19.3 ± 2.0	27.0 ± 4.6
HVal (% w/w)	<LD^*^	<LD^*^	15.7 ± 1.0	17.2 ± 0.1
HCa (% w/w)	<LD^*^	<LD^*^	0.6 ± 0.2	1.2 ± 0.2
EtOH (% w/w)	49.4 ± 9.6	30.6 ± 2.9	29.0 ± 1.7	3.2 ± 2.3
HLac (% w/w)	41.4 ± 5.1	51.5 ± 2.3	<LD^*^	<LD^*^
H_2_ (mL·g COD_in_^−1^)	<LD^*^	3.0 ± 2.3	4.8 ± 2.6	<LD^*^
CH_4_ (mL·g COD_in_^−1^)	<LD^*^	<LD^*^	<LD^*^	<LD^*^
Bioconversion metabolites (%)	63.4 ± 6.8	68.7 ± 1.3	74.5 ± 0.5	67.6 ± 0.7
Bioconversion SCFAs (%)	3.4 ± 0.3	9.4 ± 2.4	47.7 ± 5.2	64.0 ± 6.3
Bioconversion EtOH (%)	42.9 ± 3.8	33.2 ± 0.4	28.1 ± 2.7	3.2 ± 6.8
Bioconversion HLac (%)	16.8 ± 1.1	26.1 ± 0.4	<LD^*^	<LD^*^
COD removal (%)	14.2 ± 1.7	14.6 ± 0.0	13.4 ± 0.3	11.5 ± 0.5
Acidification (%)	99.0 ± 2.7	97.7 ± 0.5	99.0 ± 3.2	99.0 ± 0.3
VS removal (%)	52.3 ± 2.5	54.4 ± 1.9	60.5 ± 2.7	58.7 ± 0.9

^*^Lower than the limit of detection (LD). The selected experimental days corresponded to those in which microbial DNA analysis was conducted (see Supplementary Figure S1 for the complete longitudinal metabolite profile).

The acidic pH attained in the CSTR (pH 4.5) resulted in high HLac and EtOH accumulation, reaching concentrations of 11.7 and 13.9 g·L^−1^, respectively (Table 3). These values corresponded to an organic matter bioconversion of 16.8% into HLac and 42.9% into EtOH. HLac and EtOH have been identified as primary intermediate metabolites, whose simultaneous production has been normally related to the prevalence of heterolactic fermentation (Burgé et al., 2015; Feng et al., 2018; Wang et al., 2020). Although HLac and EtOH might be degraded into SCFAs during optimal AF devoted to carboxylate production, the low pH likely limited the acidogenic activity. This was in accordance with previous studies that determined that acidogenic bacteria are hindered at extremely low pHs (Zheng et al., 2015). Similarly, Greses et al. (2022) also observed an HLac accumulation between 2 and 13 g·L^−1^ when C-AF of fruit and vegetable waste was performed under similar conditions (25 °C and pH 4.6) but the addition of an external inoculum collected from AD. Nevertheless, an EtOH concentration lower than 1 g·L^−1^ was detected by these authors. These results revealed a significant impact of the inoculum source on the metabolic behavior of AF, since the high accumulation of EtOH in the present study evidenced the high activity of ethanologenic microorganisms when using IMOs.

**Figure 2:**
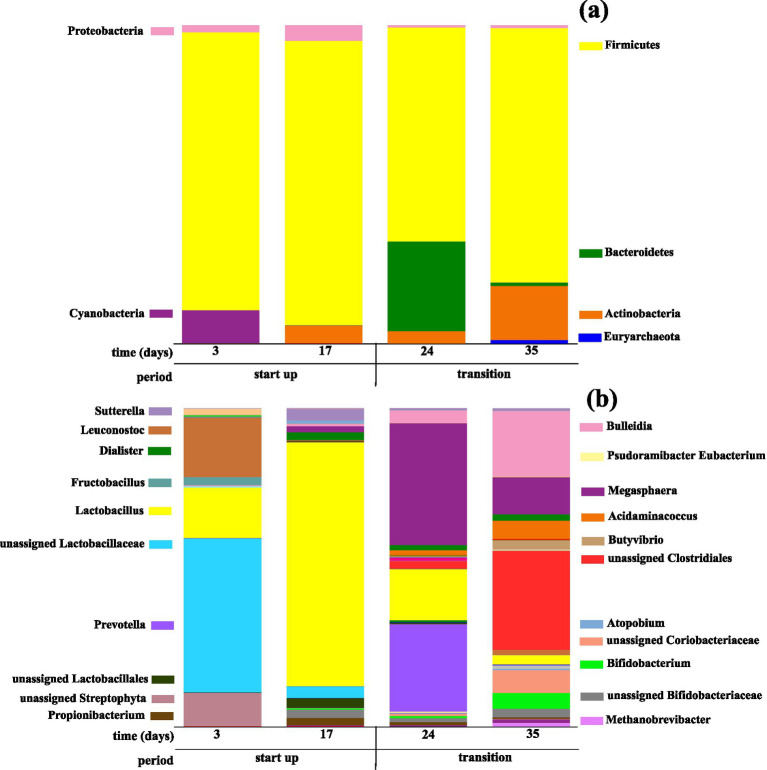
Relative abundance of bacteria and archaea were identified throughout the experiment at **(a)** phylum and **(b)** genus levels. Microorganisms with relative abundance lower than 1% have been excluded from the plot legend.

Microbial community analysis revealed that the initial days (day 3) were characterized by microorganisms belonging to the Firmicutes phylum (89.3%) (Figure 2a), such as the Lactobacillaceae family (48.4%), *Leuconostoc* (18.6%), *Lactobacillus* (15.8%), and *Fructobacillus* (2.6%) genera (Figure 2b) within the order Lactobacillales. To a minor extent, sequences assigned to the Streptophyta order (10.4%) (Cyanobacteria phylum) were also detected (Figure 2) as substrate-derived signals, likely reflecting the presence of plant-derived chloroplast DNA in the AGW.

Lactobacillaceae, *Leuconostoc*, *Lactobacillus,* and *Fructobacillus* have been described as lactic acid bacteria (LAB), which are commonly found on the surface of fruits and vegetables and in raw food waste (Leff and Fierer, 2013; Mohamed et al., 2023; Ngouénam et al., 2021; Zhang et al., 2022). These microorganisms have been reported to thrive at acidic pH (Feng et al., 2018), which was consistent with the pH drop determined at this point of the fermentation (pH 4.5). Indeed, these microorganisms are known to exhibit a high affinity for the degradation of fructose and glucose with the concomitant release of EtOH and HLac as main end products. This trend was in accordance with the high content of fructose and glucose determined in the AGW (Table 1) and strongly correlates with the high concentrations of these metabolites in the effluent (13.9 and 11.7 g·L^−1^, respectively). Moreover, these LAB are hypothesized to have also contributed to HAc production (2.1 g·L^−1^) since their enrichment has been correlated with HAc synthesis from fructose and glucose degradation (Mohamed et al., 2023; Mutungwazi et al., 2023; Pérez-Rangel et al., 2023). The presence of these bacteria, along with the metabolite profile, revealed the potential of IMOs to produce value-added metabolites (HLac and EtOH) beyond SCFAs without the need to rely on external microbial supplementation.

Once the pH control implementation started, the process pH was maintained at 5.9 on day 17. Consequently, the EtOH concentration slightly decreased (10.8 g·L^−1^), while the concentrations of HLac (18.2 g·L^−1^), HAc (4.3 g·L^−1^), and HPro (1.6 g·L^−1^) increased, reaching a total metabolite concentration of 35.4 g L^−1^ (Table 3). Similar results were observed by Wu et al. (2017), who found EtOH as the main metabolite at pH 4 when performing AF of fruits and vegetables using a conventional inoculum and a prevalence of HAc with a concomitant EtOH decrease at pHs between 5.0 and 6.0. Although only a slight metabolite outcome change was observed, the microbial profile underwent a complete transformation. A significant increase in the relative abundance of the *Lactobacillus* genus (76.5%) took place at the expense of other members of the Lactobacillaceae family (Figure 2b). The SIMPER analysis revealed that the microbial communities identified at days 3 and 17 exhibited a dissimilarity of 78.5%. This fact was mainly attributed to *Lactobacillus*, which described 39.6% of this microbial difference (Supplementary Table S2) due to a significant relative abundance increase from 15.8% (day 3) to 76.5% (day 17). The dominance of *Lactobacillus* within the microbiome on day 17 contributed to the high HLac production reached for this sampling point, agreeing with the high metabolic affinity of *Lactobacillus* for the sugars present in AGW (Tang et al., 2017). These results confirmed that *Lactobacillus* was primarily responsible for the observed shift in the metabolite profile at this stage (day 17). This result was confirmed by the CCA plot (Supplementary Figure S3), where the start-up samples (days 3 and 17) clustered in the positive quadrant of CCA 1, showing a strong correlation with the vectors for HLac and EtOH. This ordination confirms that the initial phase was governed by active heterolactic fermentation driven by the indigenous LAB community. Self-AF day 3 scored negatively on CCA 3 and was associated with *Leuconostoc* and unassigned Lactobacillaceae, which is consistent with the dominance of diverse heterofermentative LAB at this stage. In contrast, self-AF day 17 scored positively on CCA 3, aligned with *Lactobacillus* and *Propionibacterium*, indicating a rapid shift toward *Lactobacillus*-driven fermentation. The isolated positioning of *Propionibacterium* in the upper region of CCA 2 and CCA 3 suggested that this genus played a transient role during the early enrichment, separate from the main LAB cluster.

The SIMPER analysis also showed that the total discrepancy between samples was covered up to 81.5% when unassigned Lactobacillaceae and *Leuconostoc* were included, since these bacteria were not detected on day 17. *Leuconostoc* and other members within the Lactobacillaceae family are well-known acidic bacteria (pH < 5) (Feng et al., 2018), which might justify their disappearance when the pH increased to 5.9 and confirmed a significant influence of pH variations on the microbial rearrangement within the microbial systems.

The pH adjustment also enabled the development of microorganisms such as *Sutterella* (3.7%), *Dialister* (2.4%), *Propionibacterium* (2.3%), *Megasphaera* (1.9%), and members within the Bifidobacteriaceae family (2.6%). The observed accumulation of HAc was consistent with the enrichment of *Sutterella*, since this metabolite has been found as one of the main end products of glucose metabolization by these bacteria (Xu et al., 2023). *Megasphaera* has been previously associated with HAc and HPro production through C-AF of sugar-rich feedstock and lactate at pH 6 (Feng et al., 2018), suggesting a similar metabolic role in the present investigation. Similarly, the *Dialister* genus was related to HAc and HPro production from carbohydrate fermentation using an external inoculum addition (Lin et al., 2021; Luo et al., 2024), which is in agreement with the metabolite profile attained herein.

The presence of members from the Bifidobacteriaceae family was also relevant when the pH increased, as these bacteria have low tolerance to extremely acidic pH (Wu et al., 2015). As previously detailed, Bifidobacteriaceae have been described as fermentative bacteria able to degrade glucose into HAc and HLac (Cieciura-Włoch et al., 2020). Hence, the enrichment of these bacteria is likely associated with an increase in the concentration of these metabolites. The production of HPro strongly correlated with an increase in the relative abundance of members of the *Propionibacterium* genus, which is in line with previous studies that determined *Propionibacterium* as HPro-producers through the lactic acid pathway (Li et al., 2014; Zheng et al., 2022).

The high concentration of primary intermediate metabolites (HLac and EtOH), along with the production of HAc and HPro observed in the initial days, demonstrated their synthesis feasibility under pH ranges between 4.5 and 6 by using IMOs. It can be thus concluded that despite not using an external inoculum, the feedstock contained common acidogenic bacteria that likely drove the accumulation of relevant products.

#### 3.2.2 Transition

On day 24, an increase in SCFA bioconversion from 9.4 to 47.0% was determined, reaching a 74.5% bioconversion of organic matter into total metabolites (Table 3). In this transition state, when a pH of 6 was attained, a shift in metabolite distribution became evident as HLac was completely consumed and the EtOH concentration started to decrease, giving rise to the synthesis of SCFAs (Table 3; Supplementary Figure S2). A change in pH alters the proton balance in the culture broth, thereby resulting in a metabolism rearrangement (Zhao et al., 2022). Extremely low extracellular pH can lead to the accumulation of protons (H^+^) inside the cells, inducing a drop in the intracellular pH. Under these conditions, only acid-resistant microorganisms are able to survive. This is the case of LAB. This type of bacteria can overexpress the H^+^-ATPase, which is a cell membrane protein responsible for pumping H^+^ out of the cell, by consuming ATP when the intracellular pH drops (Yang et al., 2024). At such a low pH, bacteria that do not exhibit strong H^+^-ATPase activity were not able to survive. As the conditions were less restrictive when the pH was increased to 6, this mediated the development of a wider number of bacteria. Although pH oscillation remained low, the SIMPER analysis (Supplementary Table S2) confirmed a significant distinction between samples collected on day 17 and day 24, reaching a dissimilarity percentage of 74.9% (Table 3). Microorganisms belonging to *Lactobacillus*, *Megasphaera,* and *Prevotella* contributed 86.2% to the overall percentage.

The development of SCFA producers, such as *Prevotella* (27.4%) and *Megasphaera* (38.2%), observed on experimental day 24 (Figure 2b), together with LAB (*Lactobacillus*) reduction, is highly consistent with the influence of these microorganisms in the metabolite profile variation determined in the transition period. SCFA (HBu, HPro, HAc, and HVal) concentration increased (Table 3), while HLac was totally exhausted, and EtOH concentration decreased (from 10.4 to 9.1 g·L^−1^). As explained in the previous section, the *Megasphaera* and *Prevotella* genera have been associated with the synthesis of organic acids. Hence, the identified microorganisms might have contributed to the disappearance of HLac and also to the significant accumulation of SCFAs observed on day 24. These results were further supported by the CCA (Supplementary Figure S3). Along the first axis (CCA 1), the transition samples (days 24 and 35) clearly migrated away from the LAB-dominated cluster toward the center of the plot. In the CCA 1–CCA 2 graph (Supplementary Figure S3a), *Megasphaera* and *Prevotella* were located in proximity to the pH and HPro, confirming that the pH increase favored the emergence of these genera and subsequent increase in HPro (Table 3). Supplementary Figure S3b successfully captures the temporal succession within this transition. Self-AF maintained a positive CCA 3 score, consistent with residual LAB activity, whereas self-AF day 35 migrated toward the center of axis CCA 3. This migration reflects the progressive displacement of primary fermenters and the consolidation of unassigned Clostridiales, Bifidobacteriaceae, and Coriobacteriaceae. Additionally, the intermediate positioning of *Acidaminococcus* near the transition zone in CCA 1 further suggests its active involvement during this metabolic reshaping.

At the end of the transition period (day 35), EtOH exhibited a considerable decrease (1.0 g·L^−1^), with the metabolite pool mainly composed of SCFAs. Concomitantly with the EtOH consumption, the specific bioconversion into SCFAs increased from 47.7 to 64.0% (Table 3). At this point, HAc was the most abundant SCFA (11.6 g·L^−1^), followed by HBu (8.6 g·L^−1^). Similar results were obtained by Zheng et al. (2015), who reported that HAc (11 g·L^−1^) and HBu (6 g·L^−1^) prevailed when performing AF of FVW at pH between 5 and 6 when employing an external inoculum from AD. Bolaji and Dionisi (2017) also observed that HAc (7.3 g·L^−1^) and HBu (3.8 g·L^−1^) were the dominant metabolites when performing C-AF of vegetables at pH 6. These previous studies strongly support the significant effect of pH on metabolite profile when using carbohydrate-rich residues as feedstock.

According to the SIMPER analysis, 71.6% dissimilarity was found between days 24 and 35 of the AF process (Supplementary Table S2), indicating that a period longer than an HRT was required to reach AF stability since pH remained stable from day 17, but the microbiome showed significant differences. The high dissimilarity was mainly associated with microorganisms belonging to the Clostridiales order, *Megasphaera, Prevotella*, *Bulleidia,* and *Lactobacillus*. Since Clostridiales and *Bulleidia* have been described as sugar consumers to produce HBu and HAc (Sun et al., 2023; Gulhane et al., 2017), these bacteria may be related to the high accumulation of those SCFAs registered for that fermentation period (Table 3; Supplementary Figure S1). The complete disappearance of *Prevotella*, simultaneously occurring with the decline of EtOH concentration, was also relevant. Liu et al. (2023) found a negative correlation between *Bulleidia* and *Prevotella*, indicating that their co-existence is hampered at pH between 6 and 6.5. These observations suggested a consistent correlation between the enrichment of *Bulleidia* and *Prevotella* and the accumulation of SCFAs and EtOH, respectively. Furthermore, the archaeal community, which remained undetected during the initial start-up (days 3 and 17) and early transition period (day 24), emerged on day 35. Specifically, archaea belonging to the *Methanobrevibacter* genus were detected for the first time in the self-AF reactor (0.9% relative abundance), which did not lead to significant CH_4_ production. Nevertheless, this marginal archaeal development did not disrupt the predominant fermentative activity.

#### 3.2.3 Steady state of self-AF

Once the self-AF reached the steady state (day 91), the bacterial community slightly evolved as a result of moving from the transition to the stable period (Figure 2: transition and Figure 1: self-AF). Clostridiales order (51.8%), Bifidobacteriaceae family (21.4%), and *Bulleidia* genus (7.8%) dominated the microbiome in self-AF. The SIMPER analysis (Supplementary Table S2) identified Clostridiales and Bifidobacteriaceae as the most significant microorganisms contributing to the dissimilarity between day 35 and the steady state of the self-AF, accounting for 43.2% of the cumulative variation. However, the overall dissimilarity (44.7%) was not as high as that calculated for earlier stages, confirming that the microbiome was close to stability on day 35, but a period longer than three HRTs was required to establish a stable SCFA-producing community. Concurrently, the archaeal community (*Methanobrevibacter*) detected on day 35 was reduced to a marginal relative abundance of 0.3%.

In this fermentation period, the high accumulation of HAc, HBu, and HCa was likely a consequence of the increase in the relative abundance of Clostridiales from 31.0% on day 35 (Figure 2) to 51.8% in the steady state (Figure 1). An increase in Bifidobacteriaceae from 2.6 to 21.7% was also highly consistent with this accumulation (Cieciura-Włoch et al., 2020). This microbial community shift was clearly reflected in the CCA (Supplementary Figure S3), as the steady-state sample (day 91) was strongly related to the vectors corresponding to HAc, HBu, and HCa along the negative regions of CCA 1 and CCA 2 (Supplementary Figure S3a) and closely associated with unassigned Clostridiales and Bifidobacteriaceae. Furthermore, the CCA 1–CCA 3 plot (Supplementary Figure S3b) revealed that the self-AF steady state was distinctly positioned in the positive region of CCA 3, specifically associated with *Bifidobacterium* and *Bulleidia*, confirming that these taxa, alongside unassigned Clostridiales and Bifidobacteriaceae, contributed collectively to the SCFA profile attained at the steady state.

Similarly, Gonçalves et al. (2024) reported a Clostridiales increase when C-AF of AGW at 25 °C was subjected to a pH increase from 5.8 to 6.2, giving rise to a predominance of HBu and HAc. These results supported the major effect of pH on the dynamics of the IMO profile and their active metabolic routes. Nevertheless, the continuous addition of alkali reagents required to maintain this specific pH implies a significant operational cost for industrial upscaling (Aboudi et al., 2023). To mitigate these economic constraints, co-fermentation of AGW with nitrogen-rich substrates (e.g., sewage sludge) is proposed due to their improved buffering capacity (Lanfranchi et al., 2022). These co-substrates, therefore, could provide natural alkalinity that hampers acidification, reducing the dependence on external chemical reagents.

Ultimately, it can be concluded that a pH control could be used to induce a microbial specialization toward SCFA production, highlighting the great capacity of the IMOs to transform the AGW into value-added metabolites. The metabolite profile shifts in response to pH changes further corroborate the microbiome’s versatility to produce a wide range of value-added compounds under different conditions.

## 4 Conclusion

High AF efficiencies demonstrated the capacity of microorganisms naturally present in the feedstock to produce added-value chemicals. The similar metabolite and bacterial profile observed in the self-AF compared with C-AF indicated that external inoculum addition was not required to successfully perform AF. Moreover, process pH was determined as a pressure factor to promote IMO enrichment and also select the specific ones based on the metabolite required. These results confirmed the use of IMOs as a promising strategy to produce a wide variety of added-value metabolites beyond SCFAs through AF, taking advantage of their outstanding metabolic versatility and strongly suppressed methanogenic activity.

## Data Availability

The data presented in this study are publicly available. The data can be found at: https://www.ncbi.nlm.nih.gov/sra, accession PRJNA907204 (SRS18431631, SRS18431630, SRS15940997, SRS15940993) and https://doi.org/10.5281/zenodo.13834172.
