# Supplementary material for: Indigenous microbiome as a key strategy for producing green chemicals
Source: Front Microbiol. 2026 Mar 27;17:1798480. doi: 10.3389/fmicb.2026.1798480 (PMC13066266; doi:10.3389/fmicb.2026.1798480)
Supplement: Supplementary file 1 [file Table_1.docx]

**Table S1.** SIMPER analysis results showing the most relevant microorganisms, contributing to the observed dissimilarity in the microbial community structure between the C-AF and self-AF.

| a) Conventional AF vs Self AF. 45.2 % dissimilarity. | | | |
| --- | --- | --- | --- |
| **Taxon** | **Average Dissimilarity (%)** | **Contribution**  **(%)** | **Cumulative**  **(%)** |
| Unassigned Bifidobacteriaceae | 11.7 | 25.9 | 25.9 |
| *Prevotella* | 8.2 | 18.1 | 44.1 |
| Unassigned Clostridiales | 4.7 | 10.5 | 54.6 |
| *Acidaminococcus* | 4.7 | 10.4 | 65.0 |
| *Bulleidia* | 3.4 | 7.6 | 72.6 |
| Unassigned Bacteroidales | 2.5 | 5.6 | 78.2 |
| *Megasphaera* | 2.5 | 5.6 | 83.7 |
| Unassigned Lachnospiraceae | 1.2 | 2.6 | 86.3 |
| *Pseudoramibacter Eubacterium* | 1.1 | 2.5 | 88.8 |
| *Bifidobacterium* | 1.0 | 2.2 | 91.0 |
| *Atopobium* | 0.9 | 2.0 | 93.0 |
| Unassigned Lactobacillales | 0.9 | 1.9 | 95.0 |
| *Methanobrevibacter* | 0.7 | 1.6 | 96.7 |
| Unassigned Veillonellaceae | 0.7 | 1.5 | 98.2 |
| *Dialister* | 0.5 | 1.0 | 99.3 |
| Unassigned Coriobacteriaceae | 0.3 | 0.8 | 100.0 |
