# Supplementary material for: Indigenous microbiome as a key strategy for producing green chemicals
Source: Front Microbiol. 2026 Mar 27;17:1798480. doi: 10.3389/fmicb.2026.1798480 (PMC13066266; doi:10.3389/fmicb.2026.1798480)
Supplement: Supplementary file 2 [file Table_2.docx]

**Table S2.** SIMPER analysis results showing the most relevant microorganisms, contributing to the observed dissimilarity in the microbial community structure between the experimental periods in self-AF.

| **a) Experimental day 3 (pH 4.5) vs day 17 (pH 5.9) (start-up): 78.4 % dissimilarity** | | | |
| --- | --- | --- | --- |
| **Taxon** | | **Contribution (%)** | **Cumulative (%)** |
| *Lactobacillus* | | 39.6 | 39.6 |
| Unassigned Lactobacillaceae | | 29.4 | 69.1 |
| *Leuconostoc* | | 12.5 | 81.5 |
| **b) Experimental day 17 (pH 5.9) vs day 24 (pH 6.0): 74.9 % dissimilarity** | | | |
| **Taxon** | | **Contribution (%)** | **Cumulative (%)** |
| *Lactobacillus* | | 41.4 | 41.4 |
| *Megasphaera* | | 24.2 | 65.5 |
| *Prevotella* | | 18.2 | 83.8 |
| **c) Experimental day 24 (pH 6.0) vs day 35 (pH 6.0) (transition): 71.6 % dissimilarity** | | | |
| **Taxon** | **Contribution (%)** | | **Cumulative (%)** |
| Unassigned Clostridiales | 23.9 | | 23.9 |
| *Megasphaera* | 20.1 | | 43.9 |
| *Prevotella* | 18.8 | | 62.7 |
| *Bulleidia* | 10.1 | | 72.8 |
| *Lactobacillus* | 9.6 | | 82.4 |
| **d) Experimental day 35 (pH 6.0) vs. steady state self-AF (pH 6.1): 44.7 % dissimilarity** | | | |
| **Taxon** | | **Contribution (%)** | **Cumulative (%)** |
| Unassigned Clostridiales | | 21.8 | 21.8 |
| Unassigned Bifidobacteriaceae | | 21.4 | 43.2 |
| *Bulleidia* | | 13.2 | 56.4 |
| *Megasphaera* | | 10.1 | 66.5 |
