# Supplementary material for: Indigenous microbiome as a key strategy for producing green chemicals
Source: Front Microbiol. 2026 Mar 27;17:1798480. doi: 10.3389/fmicb.2026.1798480 (PMC13066266; doi:10.3389/fmicb.2026.1798480)
Supplement: Supplementary file 3 [file Table_3.docx]

**Table S3.** Stability index (SI) calculated in the steady-state of C-AF and Self-AF.

|  | **C-AF** | **Self-AF** |
| --- | --- | --- |
| pH | 1.0 | 1.0 |
| TCOD (g·L^-1^) | 1.0 | 0.9 |
| SCOD (% w/w) | 1.0 | 1.0 |
| TS (g·L^-1^) | 1.0 | 1.0 |
| VS (% w/w) | 1.0 | 1.0 |
| NH_4_^+^-N (g N·L^-1^) | 0.9 | 0.8 |
| Total metabolites (g·L^-1^) | 1.0 | 1.0 |
| HAc (% w/w) | 0.9 | 0.9 |
| HPro (% w/w) | 0.9 | 0.9 |
| HBu (% w/w) | 1.0 | 1.0 |
| HVal (% w/w) | 1.0 | 0.8 |
| HCa (% w/w) | 0.8 | 0.9 |
| H_2_ (mL·g COD_in_^-1^) | <LD^*^ | 0.2 |
| CH_4_ (mL·g COD_in_^-1^) | 0.7 | <LD^*^ |

*Lower limit of detection (LD)
