# Supplementary figures and images for: Indigenous microbiome as a key strategy for producing green chemicals
Source: Front Microbiol. 2026 Mar 27;17:1798480. doi: 10.3389/fmicb.2026.1798480 (PMC13066266; doi:10.3389/fmicb.2026.1798480)

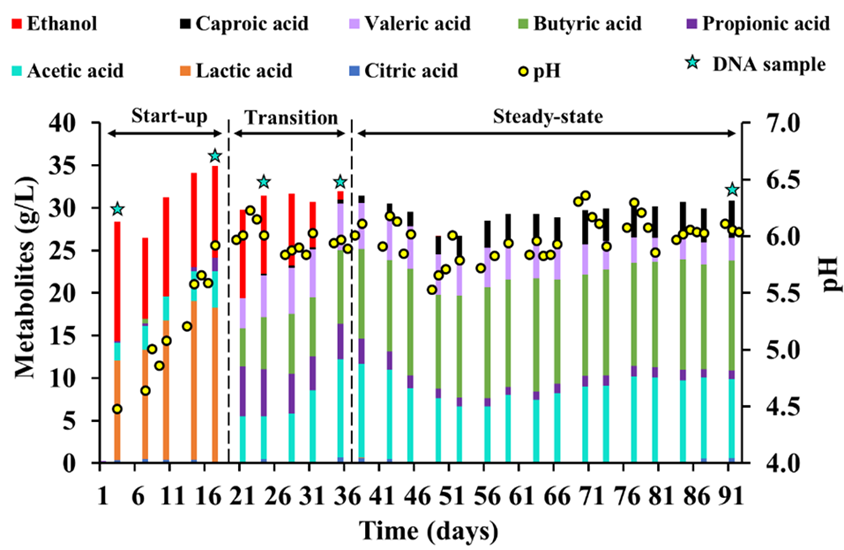


**Figure S1.** Metabolites production and profile, and pH during the self-AF of AGW.

Supplement: Supplementary file 4 [file Data_Sheet_1.docx]
