# Supplementary material for: Indigenous microbiome as a key strategy for producing green chemicals
Source: Front Microbiol. 2026 Mar 27;17:1798480. doi: 10.3389/fmicb.2026.1798480 (PMC13066266; doi:10.3389/fmicb.2026.1798480)
Supplement: Supplementary file 5 [file Data_Sheet_2.docx]

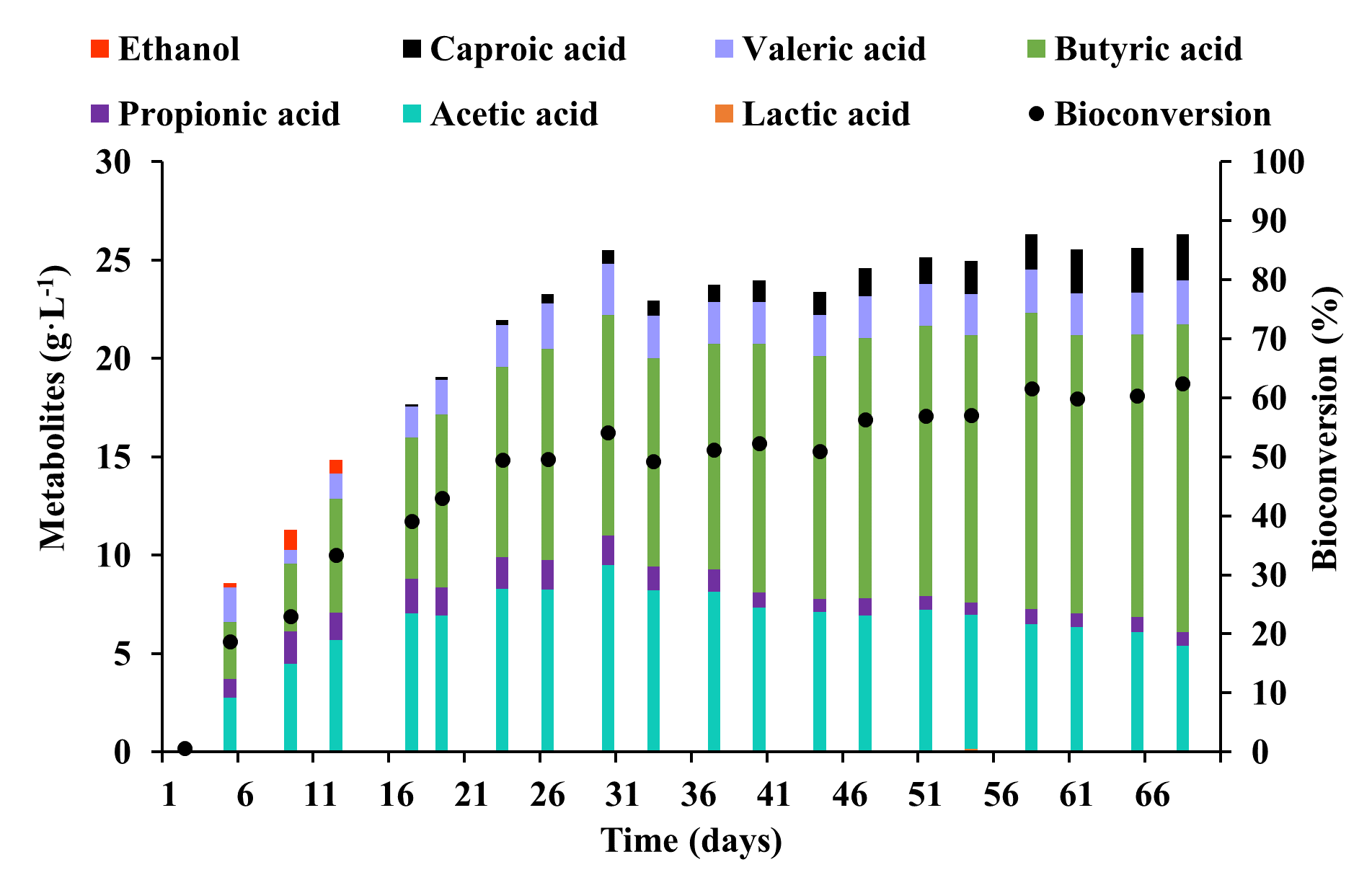


**Figure S2.** Time evolution of metabolite profile and concentration, along with bioconversion efficiency throughout the whole C-AF process.
