# Supplementary material for: Indigenous microbiome as a key strategy for producing green chemicals
Source: Front Microbiol. 2026 Mar 27;17:1798480. doi: 10.3389/fmicb.2026.1798480 (PMC13066266; doi:10.3389/fmicb.2026.1798480)
Supplement: Supplementary file 6 [file Data_Sheet_3.docx]

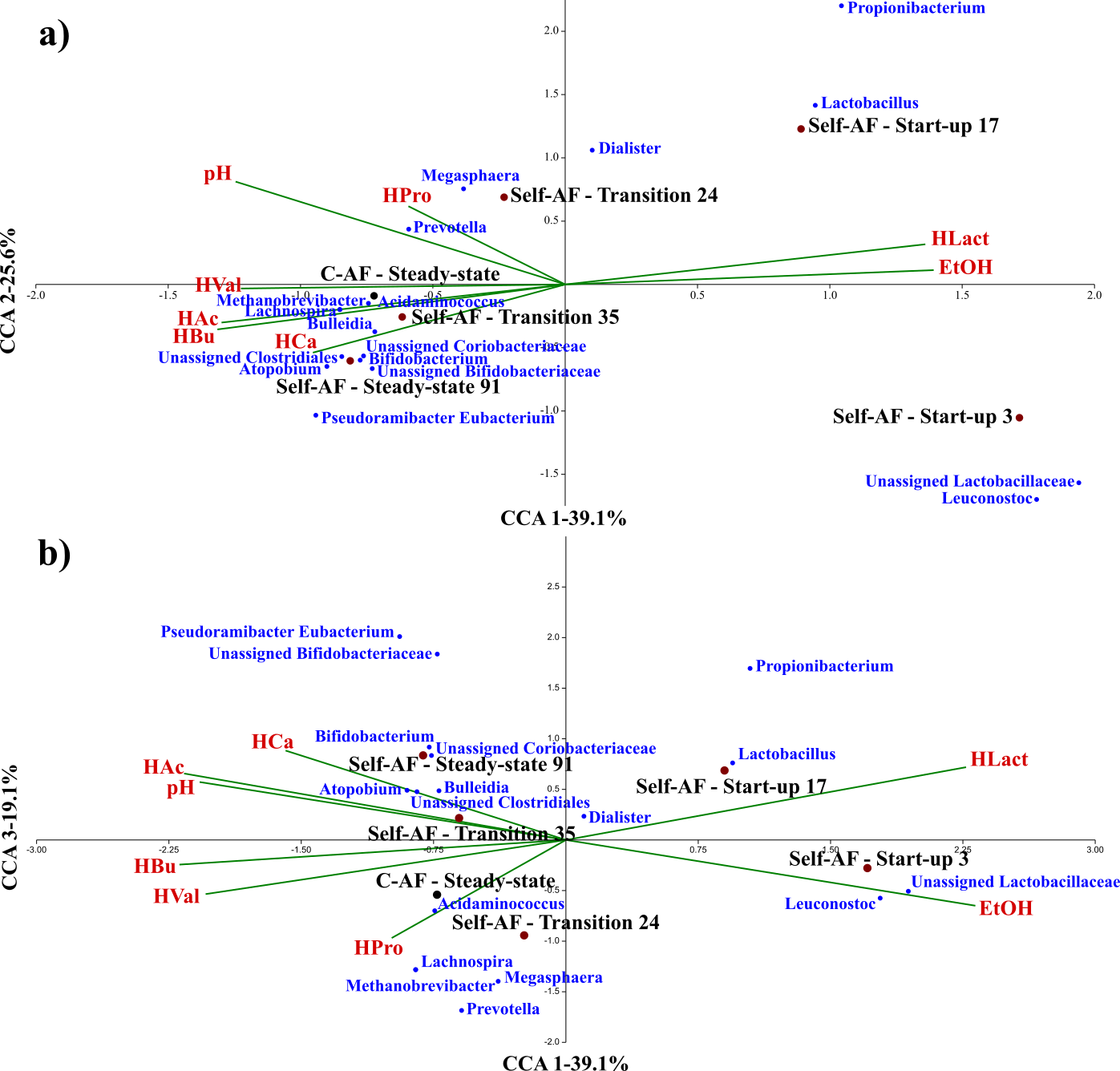


**Figure S3**. Canonical correspondence analysis ordination triplot of the Self-AF samples (days 3, 17, 24, 35 and 91) and the C-AF (steady-state). a) CCA 1 vs CCA 2, b) CCA 1 vs CCA 3.
