# Supplementary material for: Indigenous microbiome as a key strategy for producing green chemicals
Source: Front Microbiol. 2026 Mar 27;17:1798480. doi: 10.3389/fmicb.2026.1798480 (PMC13066266; doi:10.3389/fmicb.2026.1798480)
Supplement: Supplementary file 7 [file Data_Sheet_4.docx]

**
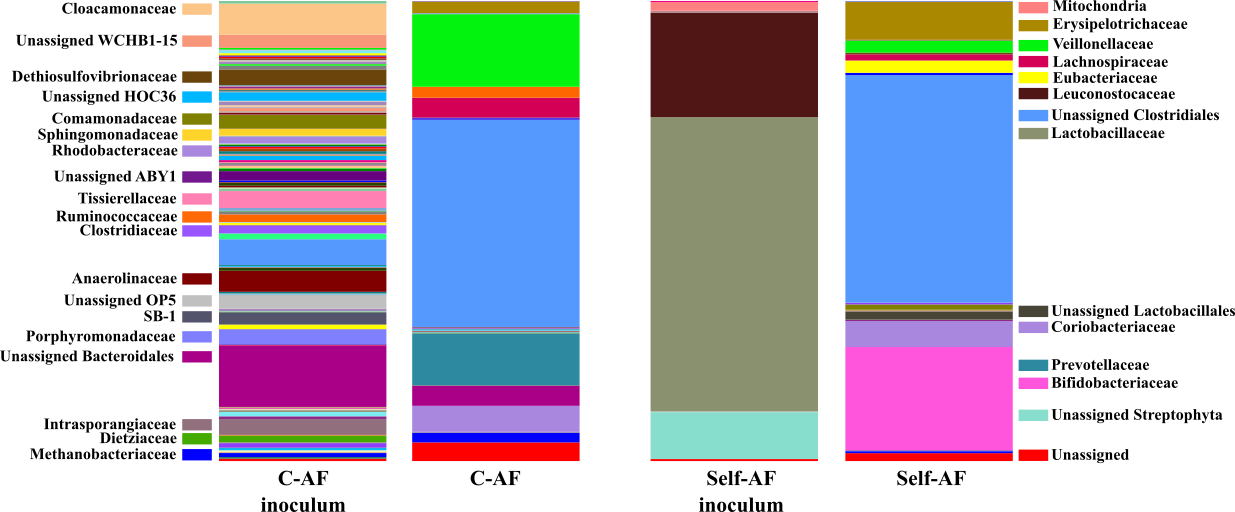
Figure S4.** Bacterial and archaeal profile determined in the inocula and the steady state (C-AF and self-AF) at family level. Microorganisms with a relative abundance lower than 1 % have been excluded from the legend.
